# The Potential of Oncolytic Virotherapy in the Treatment of Head and Neck Cancer: A Comprehensive Review

**DOI:** 10.3390/ijms252312990

**Published:** 2024-12-03

**Authors:** Francesca Spirito, Riccardo Nocini, Giorgio Mori, Massimo Albanese, Eleni A. Georgakopoulou, Gowri Sivaramakrishnan, Basel Khalil, Bruno Špiljak, Varun Surya, Deepika Mishra, Akhilanand Chaurasia

**Affiliations:** 1 Department of Clinical and Experimental Medicine, University of Foggia, 71122 Foggia, Italy; giorgio.mori@unifg.it; 2 Department of Surgical Sciences, Dentistry, Gynaecology and Paediatrics, University of Verona, 37134 Verona, Italy; riccardo.nocini@gmail.com (R.N.); massimo.albanese@univr.it (M.A.); 3 Department of Histology and Embryology, Medical School, National and Kapodistrian University of Athens, 11527 Athens, Greece; eageorgakopoulou@gmail.com; 4 Bahrain Defence Force Royal Medical Services, Riffa 28743, Bahrain; gowri.sivaramakrishnan@gmail.com; 5 Department of Basic Sciences, Faculty of Dentistry, University of Damascus, Damascus 30621, Syria; 6 Department of Oral Medicine, School of Dental Medicine, University of Zagreb, 10000 Zagreb, Croatia; bspiljak@sfzg.unizg.hr; 7 Department of Oral Pathology and Microbiology, Centre for Dental Educationand Research, All India Institute of Medical Sciences, New Delhi 110029, India; drvarunsuryamds@gmail.com (V.S.); deepika1904@gmail.com (D.M.); 8 Department of Oral Medicine and Radiology, King George’s Medical University, Lucknow 226003, India; chaurasiaakhilanand49@gmail.com

**Keywords:** head and neck cancer, squamous cell carcinoma, oncolytic virus, therapy

## Abstract

Head and neck cancer (HNC) represents a challenging oncological entity with significant morbidity and mortality rates. Despite advances in conventional therapies, including surgery, chemotherapy, and radiation therapy, the overall survival rates for advanced HNC remain suboptimal. In recent years, the emerging field of oncolytic virotherapy has gained attention as a promising therapeutic approach for various malignancies, including HNC. This review provides a comprehensive overview of the current understanding of oncolytic viruses (Ovs) in the context of HNC treatment, including their mechanisms of action, preclinical and clinical studies, challenges, and future directions. Future oncolytic virotherapy focuses on improving delivery and specificity through nanoparticle carriers and genetic modifications to enhance tumor targeting and immune response. Combining different OVs and integrating them with immunotherapies, such as checkpoint inhibitors, could overcome tumor resistance and improve outcomes. Personalized approaches and rigorous clinical trials are key to ensuring the safety and effectiveness of virotherapy in treating HNC.

## 1. Introduction

### 1.1. Head and Neck Squamous Cell Carcinoma: Epidemiology, Diagnosis, and Treatment Options

Head and neck cancer is the sixth most common cancer globally, accounting for approximately 6% of all malignancies [1]. Squamous cell carcinoma (SCC) represents about 90% of all head and neck cancers [2], typically arising in the oral cavity, nasal cavity, paranasal sinuses, pharynx, and larynx [3]. The head and neck region’s intricate anatomical and physiological features contribute to the significant heterogeneity observed in head and neck squamous cell carcinoma (HNSCC). Over 60% of patients are initially diagnosed with advanced-stage disease, and despite comprehensive treatment, the rates of metastasis or recurrence range from 40% to 60% [4,5], with a five-year rate that can vary widely from 10% to 82%, depending on factors such as disease stage, patient age, race, comorbidities, and tumor location [6,7,8]. The primary treatments for HNSCC include surgery [9], radiation therapy, and chemotherapy, often used in various combinations [10,11]. Surgery remains the standard initial treatment for most oral cancers [12], with a well-established history of efficacy. Ionizing radiation has emerged as a critical nonsurgical treatment option, frequently used alongside surgery, particularly in advanced cases. In instances of local or regional recurrence, treatment strategies range from salvage surgery to radiation combined with chemotherapy or chemotherapy alone if the disease is beyond salvage [13]. Additional treatments for HNCs include targeted therapy and immunotherapy [14] that leverage the patient’s immune system to stimulate antitumor responses, target and destroy cancer cells, and counteract tumor-induced immune suppression [15].

### 1.2. Oncolytic Virotherapy: A Resurgent Strategy in Cancer Treatment

Oncolytic virotherapy has emerged as a promising new treatment avenue. The historical link between microbial infections and spontaneous tumor regressions has been well-documented, with early 20th-century reports highlighting viral infections leading to temporary remissions in leukemia patients [16]. Although the concept of using viruses to treat cancer dates back to the pre-1950s, the lack of consistent efficacy and safety led to a decline in virotherapy research, with other treatments such as chemotherapy and radiation therapy becoming more prevalent [17]. However, the field of oncolytic virotherapy experienced a resurgence in the late 20th century, driven by advancements in molecular biotechnology and a better understanding of immune system interactions [18].

Advances in gene engineering have allowed for the development of virus mutants that are deficient in harmful genes while incorporating foreign genes to enhance oncolytic potential and stimulate immune responses, thereby improving tumor immunity [19]. Herpes simplex virus type 1 (HSV-1) was the first engineered virus tested for safety and efficacy in treating brain tumors in animal models [20]. Currently, numerous oncolytic viruses (Ovs) are undergoing clinical trials for various cancers, including brain tumors, lung cancer, melanoma, breast cancer, pancreatic cancer, sarcoma, stomach cancer, and HNSCC [21]. OVs, which selectively infect and lyse cancer cells while sparing normal cells, have become a cornerstone of this new era of cancer treatment [19]. This approach has been integrated with other therapeutic strategies, including adoptive cell therapies, monoclonal antibodies, and immune checkpoint inhibitors, which collectively have shown durable and effective clinical responses in cancer patients [22]. Additionally, conventional treatments like radiation and chemotherapy are now recognized for their immunomodulatory effects, further enhancing the potential of oncolytic virotherapy [23]. The ongoing evolution of viral vector-based cancer vaccines, gene therapies, and OVs continues to revolutionize cancer treatment, providing new hope for improved outcomes in HNSCC and other malignancies.

### 1.3. Aim

This review provides a comprehensive overview of the current understanding of OVs in the context of HNC treatment, including their mechanisms of action, preclinical and clinical studies, challenges, and future directions.

## 2. Mechanism of Action

Within the realm of immunotherapy, three overarching avenues stand out: reinvigorating anti-tumor immune surveillance, directly stimulating or blocking receptors to trigger tumor apoptosis, and precisely labeling malignant cells as targets for elimination [24,25,26,27,28,29].

The distinct advantage of oncoviral immunotherapy lies in its precise targeting of tumor cells, extending beyond just replicating ones [30,31]. OVs exhibit reduced reliance on specific receptor expression patterns, thereby mitigating the risk of mutational resistance or adaptive changes in cellular pathways. While it’s accurate that some OVs don’t depend on specific receptor patterns, many still rely on particular receptors for cell entry (e.g., adenoviruses use coxsackievirus and adenovirus receptor (CAR), coxsackieviruses use intercellular adhesion molecule 1 (ICAM-1)). These versatile viruses can either amplify existing but ineffective anti-tumor immunity or trigger a novel immune response against non-self-antigens [29,32,33].

OVs, which include various DNA and RNA viruses, can selectively infect and replicate within tumor cells, causing their destruction while sparing surrounding healthy tissue. OVs are generally classified into three categories:

1. Natural OVs are viruses that inherently replicate within tumor cells without any modifications.
2. Second-generation Ovs are modified viruses, achieved through genetic alterations such as the deletion of viral genes, use of transcriptional elements as promoters or enhancers, or changes to viral surface proteins.
3. Third-generation OVs are genetically engineered viruses designed to express therapeutic genes like granulocyte-macrophage colony-stimulating factor (GM-CSF), combining multiple benefits to enhance their oncolytic and immune-stimulating effects.

Each type of OV has distinct therapeutic applications and challenges [34]. For instance, viruses engineered with cytokine genes, such as granulocyte-macrophage colony-stimulating factor (GM-CSF), are often used to enhance immunogenicity. These viruses can stimulate a stronger immune response against tumor cells, potentially improving treatment outcomes [35]. However, this approach may require careful dose management due to the heightened risks of immune response, which can lead to adverse effects such as cytokine release syndrome (CRS) and autoimmune reactions [36].

This genetic tweaking boosts their affinity for cancerous tissues and diminishes their virulence towards non-neoplastic host cells. Consequently, these viruses induce a proinflammatory milieu by promoting the release and recognition of antigens, triggering a cascade of immune responses aimed at overcoming the immune evasion strategies employed by malignant cells [37]. Moreover, OVs are designed to exploit the tumor’s immune-tolerant mechanisms, facilitating viral infection and the destruction of cells that lack protection from the immune system [37]. This strategic approach sets off a potential chain reaction, wherein the viruses transfer between cancerous cells, leading to further immune activation and ultimately amplifying the anti-cancer immune response [38].

The cancer immunoediting process unfolds in three distinct stages: elimination, equilibrium, and escape. During the elimination phase, malignant cells are promptly detected and cleared by the immune system, often involving lymphocytes and glycoproteins that sculpt the tumor. This phase transitions into equilibrium, where a delicate balance is maintained between the immune response and the tumor’s growth. Finally, the tumor may achieve immune evasion through altered transcription, marking the onset of the escape phase [33,39,40].

In the elimination phase, a relentless onslaught of T-cell-mediated eradication targets malignant cells. This process involves an array of effector responses orchestrated by CD8+ T-cells, γδ T-cell subsets, and NK cells. Additionally, crucial macromolecules such as IFNγ, perforin, and TNF (tumor necrosis factor)-related apoptosis-inducing ligands contribute to the robust immune assault on cancerous cells [27,32,41,42,43,44].

Like other forms of immunotherapy, OVs exhibit a multifaceted mechanism of action, exerting both direct and indirect toxic effects on tumor cells. These effects encompass autolysis, the precise targeting and homing of immune cells to tumor sites, disruption of the tumor’s vascular supply, and the enhancement of other complementary anti-cancer treatments [37,45]. This intricate interplay of mechanisms underscores the versatility and potency of OVs as a promising tool in the fight against cancer.

One method of inducing toxic injury is through direct cell lysis, facilitated by traditional antiviral machinery. This process is hypothesized to be dose-dependent, with remarkable tolerability even at elevated doses [37,46]. For example, when cells become infected, they can trigger a response via interferons or Toll-like receptors by transcribing antigens, which are subsequently presented on the cell surface or detected by intracellular components of Toll-like receptors. These antigens, known as pathogen-associated molecular patterns (PAMPs), encompass various viral components such as the capsid, nucleic acids, or proteins.

Recognition of virally infected cells by the immune system initiates a cascade involving factors like TNF and IFN (interferon)-related factors, along with retinoic acid-inducible gene 1 (RIG-1), stimulating the JAK (Janus kinase)/STAT (signal transducer and activator of transcription) pathway. This pathway provides positive feedback to IFN, activating protein kinase R (PKR). PKR, in turn, senses intracellular viral material, halting protein transcription and ultimately promoting apoptosis (programmed cell death) and viral clearance [47]. This orchestrated series of events highlights the intricate interplay between viral infection and the immune response, crucial for combating viral pathogens and potentially beneficial in the context of oncolytic virotherapy.

It is important to note that not all cancer cells exhibit inactivated or defective interferon production or response pathways. While defects in these pathways can create environments favorable to OV selectivity, many tumors retain intact antiviral mechanisms. OVs can effectively target even tumors with functional interferon and PKR pathways, leveraging these pathways to enhance immune system anti-tumor effects. When an OV infects tumor cells, it triggers the production of interferons, which are potent immune-stimulating proteins. These interferons activate immune cells such as natural killer cells and T-cells, empowering them to recognize and eliminate tumor cells more efficiently [48]. Additionally, interferons induce inflammation within the tumor microenvironment, exposing tumor antigens to the immune system. As the OV replicates and lyses infected tumor cells, it releases these tumor antigens, further amplifying the immune response1. This synergistic interplay between interferon-mediated immune activation and the release of tumor antigens results in a robust and targeted immune attack against the tumor [48].

Moreover, infected cells exhibit the transcription of cytokines and other proinflammatory signaling peptides [37,49]. For instance, molecules like HMGB1, calreticulin, and viral or cellular DNA can be released into the tumor microenvironment, eliciting the recruitment of immune cells [50,51,52]. Some of these anti-viral signaling mechanisms involve the selective upregulation of peptides and small interfering RNAs (siRNAs). Importantly, these responses are not typically observed in non-tumor host tissue cells [53].

Another noteworthy mechanism, exemplified by the coxsackievirus targeting non-small cell lung cancer, involves the proliferation of specific viral antigens that disrupt essential cell survival pathways, as demonstrated by the B3 antigen disrupting the ERK/MEK pathway [54]. Cytometric analyses have further revealed the upregulation of immunotherapeutic targets, such as CTLA-4, in tumor-infiltrating T-cells. This observation suggests a potential role for OVs in neo-adjuvant/adjuvant treatment alongside systemic immunotherapies [55].

Tumor specificity in OVs arises from a diverse array of mechanisms. One pivotal mechanism involves the presence of defective antiviral defense mechanisms within tumor cells.

In normal cells, viral protein synthesis faces inhibition due to activated interferon and the protein kinase pathway (PKR). When a virus infiltrates the body, interferons kick into action, subsequently activating the PKR pathway, which effectively suppresses viral protein synthesis.

However, in cancer cells, the story takes a different turn. Interferons become inactivated while the Ras pathways awaken (Ras has a knack for inactivating the PKR pathway). The result? A defective antiviral response within cancer cells.

Some of these naturally occurring OVs operate via these mechanisms. Notable examples include the Newcastle disease virus, reovirus, vaccinia virus, and vesicular stomatitis virus [56,57,58]. Certain tumor cells boast overexpressed or mutated receptors, unique to cancer cells and absent in normal ones. Through these receptors, OVs can penetrate tumor cells, replicate within them, and selectively eliminate them while sparing healthy cells [59,60,61].

Another avenue for achieving tumor specificity involves incorporating OVs with gene promoters that thrive in specific tumors [62]. For instance, papillomavirus promoters exhibit high specificity for oral cancer cells. When an HSV-1 strain is armed with papillomavirus promoters, it skillfully homes in on oral cancer cells [63,64].

Within the tumor microenvironment, proteases play a crucial role. For example, the reovirus relies on the proteolytic breakdown of its outer viral coat into subviral particles for successful infection. Tumors with an abundance of proteases facilitate virus activation within their cells.

In this intricate dance of molecular interactions, OVs wield their specificity as a potent weapon against cancer, navigating the delicate balance between destruction and preservation [65,66,67].

## 3. Clinical Trials on Oncolytic Viruses for HNSCC

Clinical trials involving OVs are advancing the potential of this innovative treatment. These trials are designed to evaluate the safety, efficacy, and optimal application of OVs in HNSCC patients, who often have limited options due to the aggressive nature of the disease [68]. Phase I trials primarily focus on determining the safety and appropriate dosing of OVs, enrolling small groups of patients who have not responded to conventional treatments. These initial studies are critical for identifying any adverse effects and establishing a foundation for further research [69]. Phase II trials expand the patient population to assess the efficacy of the OVs more comprehensively. Researchers monitor tumor response, progression-free survival, and overall survival rates and continue to track safety. These trials provide valuable insights into the therapeutic potential of OVs, helping to identify which patient populations may benefit the most [70]. Notably, these trials often include the investigation of biomarkers to predict patient response, aiming for a more personalized treatment approach. Phase III trials involve larger groups of patients and compare the effectiveness of the OV against standard treatments. This phase is crucial for determining the OV’s relative efficacy and ensuring its safety profile is robust. Successful Phase III trials can lead to regulatory approval and the integration of OVs into standard HNSCC treatment protocols [69].

Clinical trials on oncolytic virotherapy face several challenges, including limited efficacy and tumor specificity, where the virus may not effectively target all cancer cells. The patient’s immune system can neutralize the virus before it acts, and delivering the virus to the tumor site is often difficult. Safety concerns arise from the risk of unintended infection and adverse reactions, and there are complex regulatory and ethical issues to navigate. Patient responses vary widely, complicating treatment standardization, and combination therapies can lead to adverse interactions. High costs limit accessibility, and the long-term effects remain uncertain due to limited clinical data, necessitating further research to ensure safety and efficacy [71]. Current clinical trials for HNSCC oncolytic virotherapies were identified by querying the ClinicalTrials.gov database. Of the included trials, four were terminated due to a poor recruitment rate. No phase 3 or 4 trials were identified. Further details on the trials are presented in Table 1:

Only one trial had published data that demonstrated the combination of T-VEC and pembrolizumab showed a tolerable safety profile in HNSCC. The efficacy of the combination was comparable to that of pembrolizumab monotherapy in historical HNSCC studies. The Phase III part of this study was not continued [72]. The other trials identified in Table 1 do not have any published data related to the safety or efficacy of these therapies. These trials are at an initial stage, and further studies are needed to provide any conclusive evidence. Overall, ongoing clinical trials are essential for advancing the use of OVs in HNSCC, providing critical data that can lead to improved treatment strategies and better patient outcomes. As research progresses, OVs have the potential to become a standard, effective treatment option for HNSCC, offering hope to patients with this challenging cancer.

## 4. Emerging Oncolytic Viruses with Potential for HNSCC Treatment

Despite advances in conventional therapies such as surgery, chemotherapy, and radiation, the prognosis for patients with advanced HNSCC remains poor, underscoring the urgent need for novel treatment modalities. Oncolytic virotherapy offers a potentially transformative approach to address this unmet clinical need by exploiting viruses’ ability to selectively infect and replicate within tumor cells, leading to their destruction through a process known as oncolysis [73]. While commercially available oncolytic virotherapy options specifically approved for HNSCC are currently limited, several agents have shown promise in preclinical and clinical studies for other cancer types [34]. These include talimogene laherparepvec (T-VEC), ONCOS-102, Rigvir, CAVATAK, HF-10, oncolytic vaccinia viruses, and other emerging Ovs [34]. Despite the lack of HNSCC-specific approvals, the encouraging results observed in other cancer types have sparked interest in investigating these oncolytic virotherapy options for HNSCC treatment [74,75].

- Talimogene Laherparepvec (T-VEC), marketed under the brand name Imlygic, is an oncolytic herpes simplex virus type 1 (HSV-1) that has been approved by the FDA (Food and Drug Administration) for the treatment of advanced melanoma [76]. It is administered through direct injection into melanoma lesions and works by selectively infecting and replicating within tumor cells, leading to their lysis. T-VEC also stimulates anti-tumor immune responses by releasing tumor-associated antigens, which activate immune cells to target and destroy cancer cells [76]. While T-VEC is not specifically indicated for HNSCC, preclinical and early clinical studies have shown promising results [77], prompting further investigation in this patient population.
- ONCOS-102 is an oncolytic adenovirus-based immunotherapy developed by Targovax, designed to selectively infect and kill cancer cells while also stimulating anti-tumor immune responses. It has been evaluated in clinical trials for various solid tumors, including advanced melanoma, prostate cancer, and mesothelioma [17,78]. In preclinical models, ONCOS-102 has demonstrated potent anti-tumor activity and the ability to induce systemic immune responses against cancer cells. While clinical data specific to HNSCC is limited, the promising results observed in other cancer types warrant further investigation of ONCOS-102 in HNSCC patients [79,80].
- Rigvir is an oncolytic virotherapy derived from the ECHO-7 virus, a naturally occurring enteric cytopathic human orphan (ECHO) virus. It has been approved for the treatment of melanoma in Latvia since 2004 and is commercially available in several European countries [81]. Rigvir works by selectively infecting and replicating within cancer cells, leading to their destruction through oncolysis. Additionally, Rigvir has been reported to modulate immune responses, enhancing anti-tumor immunity. Clinical studies and real-world evidence have demonstrated favorable safety profiles and efficacy outcomes in melanoma patients, including prolonged survival and reduced risk of disease progression [81,82,83]. While Rigvir has primarily been studied in melanoma, there is growing interest in exploring its potential efficacy in other cancer types, including HNSCC [80,81].
- CAVATAK, also known as Coxsackievirus A21 (CVA21), is an oncolytic virus that has shown promise in clinical trials for various cancers. While Cavatak has demonstrated activity as a monotherapy, its potential in combination with immune checkpoint inhibitors (ICIs) is being explored in clinical trials. These trials aim to evaluate the synergistic effects of Cavatak and ICIs in enhancing antitumor immune responses and improving treatment outcomes for patients with different cancer types [84,85]. While Cavatak itself is not an immune checkpoint inhibitor, combining it with ICIs represents a strategy to harness both its oncolytic properties and the immunomodulatory effects of ICIs to enhance overall therapeutic efficacy [84].
- HF-10 is a type of oncolytic virus derived from herpes simplex virus type 1 (HSV-1). It has shown promising results in clinical trials for various types of cancer, including melanoma, head and neck cancer, and pancreatic cancer [34,73,86]. HF-10 works by selectively infecting and destroying cancer cells while sparing healthy cells. Clinical studies have demonstrated its safety and efficacy as a monotherapy. Additionally, HF-10 is being investigated in combination with other therapies, including immune checkpoint inhibitors (ICIs), to enhance its antitumor effects. The combination of HF-10 with ICIs aims to leverage the virus’s ability to stimulate an immune response against cancer cells, potentially leading to improved treatment outcomes for patients with advanced or refractory cancers [87].
- Oncolytic vaccinia virus-based therapies represent another promising approach in oncolytic virotherapy for HNSCC. The vaccinia virus, a member of the poxvirus family, has been extensively studied for its oncolytic properties and ability to selectively infect and replicate within cancer cells. These viruses can be genetically modified to enhance tumor specificity, potency, and safety [88]. Additionally, oncolytic vaccinia viruses can be armed with therapeutic transgenes to further augment their anti-tumor effects. Clinical trials investigating various oncolytic vaccinia virus-based therapies, either as monotherapy or in combination with other treatments, are underway for different cancer types, including HNSCC. Preliminary data from preclinical and early clinical studies have shown promising results, supporting further evaluation of oncolytic vaccinia virus therapies in HNSCC patients [34,73,89].

In summary, while there is not a commercially available oncolytic virotherapy specifically approved for HNSCC, several promising options are being investigated for their potential in this challenging disease. Rigvir, oncolytic vaccinia viruses, and other emerging Ovs offer unique mechanisms of action and therapeutic potential for HNSCC treatment. Further research, including large-scale clinical trials, is needed to establish the safety, efficacy, and optimal use of these oncolytic virotherapy options in HNSCC patients. Additionally, exploring combination therapies involving OVs with other treatment modalities holds promise for improving outcomes and overcoming treatment resistance in HNSCC.

## 5. T-VEC in Head and Neck Cancer

A patient with chronic leukemia who developed recurrent and metastatic cutaneous squamous cell carcinoma (CSCC) was treated with off-label T-VEC therapy [90]. The patient had previously undergone surgery for a large ulcer near his left ear, which was diagnosed as CSCC. After eight months, a new nodule in front of his left ear was discovered, which was confirmed as CSCC. The patient underwent a near-total left auriculectomy and received treatment with pembrolizumab, a checkpoint inhibitor. After four months of treatment, the patient experienced the development of two palpable left cervical lymph nodes. The patient expressed interest in off-label T-VEC treatment, which involved 11 cycles over a span of five months. The neck nodes improved with treatment, and the periauricular skin ulcer began to heal. The patient responded positively to the treatment, experiencing only a mild fever and a brief bout of cellulitis around the ear that was successfully treated with oral tedizolid. Approximately six months after the final TVEC injection, a PET scan revealed that the neck nodes had completely resolved. However, 9 months after the last T-VEC treatment, there was a slight erosion on the lower part of the periauricular ulcer, prompted by a punch biopsy that confirmed the presence of CSCC. The patient resumed T-VEC treatment, administering an extra 5 cycles every 2 weeks to the lower part of the periauricular cutaneous ulcer, which resulted in clinical improvement and no need for further treatment [90]. We have identified individuals with head and neck cancer in three trials of T-VEC. The first, which began in 2006, featured six patients with head and neck cancer and was part of a broader research project [91]. Most of them had a positive response in terms of safety to medication administration [91]. In 2010, 17 patients participated in T-VEC research, especially for patients with stage three and four head and neck cancer, and after two years, the survival rate was 82% [92].

In a 2020 trial, patients got the virus at a concentration of 10^6^ plaque-forming units (PFU)/mL (max 8 mL); a PFU represents one viral unit—that is, a virus or virus particle that can infect a cell [72].

In the 2020 research, patients received intra-tumor injections utilizing ultrasound guidance. The authors state that no injections were performed through the mouth-oral mucosa into the tumors [72]. A total of 36 patients participated in the trial, and there were no major side effects [72]. All the patients underwent concurrent immunotherapy [72]. All the patients were previously treated with traditional chemotherapy with platinum and had no response [72].

A positive response was seen in 13% of patients, which is encouraging given the advanced stage of the disease [72].

Also, despite the disease’s late state, no major side effects were seen that would be a contraindication in immunocompromised patients, as the T-VEC treatment entails the administration of a virus, even if it is inactive [72].

## 6. Combination Therapy with Oncolytic Viruses and Other Immunotherapeutic Approaches

Combining chemotherapy with oncolytic virotherapy represents a promising therapeutic strategy for cancer treatment. For complex diseases like cancer, effective therapies often arise from the integration of multiple modalities that work in synergy to enhance therapeutic outcomes [93]. One approach involves using OVs as adjuncts to conventional chemotherapy regimens, a particularly relevant strategy given that most cancer patients undergo standard chemotherapy as part of their treatment. Another approach focuses on utilizing drugs that can amplify virotherapy by inhibiting host mechanisms that suppress its effectiveness. [94]. While many OV-drug combinations have demonstrated synergistic effects, improving the efficacy compared to either treatment alone, this is not universally true. The success of these combinations can vary depending on factors such as the specific OV strain, the type of cancer being targeted, and the chemotherapy agents employed. To address these challenges, second-generation OVs are being developed with enhanced capabilities, aiming to overcome the limitations of monotherapy and optimize their use alongside specific chemotherapeutic agents [94].

It was reported that OV monotherapy had a 25% ORR, while OVs combined with ICIs had a 45% ORR for advanced melanoma treatment. OVs combined with chemotherapy (e.g., 5-fluorouracil (5-FU), paclitaxel, doxorubicin, or cyclophosphamide) and ICIs (e.g., pembrolizumab or ipilimumab) are available, and an increasing number of clinical trials are being conducted [95]. It has been reported that OVs combined with cisplatin and 5-fluorouracil in patients with recurrent head and neck cancer have achieved an 53% ORR [96].

### 6.1. Combination of Oncolytic Viruses and Chemotherapy Drugs

For OVs, many preclinical models have shown that combining chemotherapy drugs with OVs may lead to an enhanced therapeutic effect, not only for oncolytic adenoviruses but also for many other Ovs (Table 2) [97]. In fact, China approved Ad-H101 in 2005 for the treatment of head and neck cancers after phase III clinical trials showed a higher response rate for Ad-H101 plus chemotherapy with 5-FU (79–72%) compared to chemotherapy alone (40%).

#### 6.1.1. Cytotoxic Agents

Many cancer therapies today depend on cytotoxic agents that primarily work by either disrupting DNA replication or interfering with microtubule structures. Several of these agents have been rigorously evaluated in combination with OVs to enhance therapeutic outcomes or improve the effectiveness of established treatment protocols [97]. Clinical trials have revealed that attenuated OVs, such as the adenovirus ONYX-015, often exhibit limited efficacy when used alone. However, when paired with traditional cytotoxic chemotherapy agents, they tend to demonstrate improved therapeutic effects. Notably, OVs have displayed synergistic interactions with a diverse range of cytotoxic drugs, highlighting their potential in combination therapies [97].

##### DNA Crosslinking Agents and OVs

Since one of the major inhibitors of OV replication and spread is the anti-viral immune response, the immunosuppressive functions of CPA have been shown to be critical in inducing synergism with OVs.

Cyclophosphamide (CPA) enhances oncolytic virotherapy by modulating the host’s immune system rather than directly increasing viral replication in cancer cells. CPA works through three primary mechanisms: reducing preimmune immunoglobulins (like IgM) and complement activation, inhibiting innate antiviral responses, and delaying adaptive immune responses. This allows OVs like HSV-1 to infect tumors more effectively and persist longer. CPA can also inhibit the recruitment of immune cells, deplete regulatory T cells, and improve tumor sensitivity to immunotherapy, enhancing the overall anti-tumor response while potentially reducing the OV dose needed for therapy [105].

Cisplatin: Cisplatin, a platinum-based chemotherapy drug, induces DNA crosslinking, leading to cancer cell apoptosis. Since its introduction in the 1970s, it has shown strong antitumor activity and has been found to synergize with various OVs (OVs) [106]. Cisplatin enhances oncolytic virotherapy by inhibiting cytokine production, boosting viral replication, and improving cytotoxicity in several cancer models, including melanoma, non-small cell lung cancer, and pancreatic cancer [99] In combination with OVs like HSV-1, reovirus, adenoviruses, and VSV, cisplatin can potentiate the treatment, reduce drug and virus doses, and promote tumor regression in preclinical studies [99]. However, it can also interfere with immune-mediated therapies, such as IL-12-armed VSV [105].

##### Nucleoside Analogs and OVs

Gemcitabine: Gemcitabine, a cytidine analog, is used against various cancers. It is activated inside cells and inhibits DNA polymerase and ribonucleotide reductase (RR), leading to disrupted DNA synthesis. Initially developed as an antiviral agent, gemcitabine was repurposed for cancer therapy. Despite inhibiting oncolytic virus (OV) replication in some cases, it often enhances overall antitumor effects when combined with OVs. Synergistic interactions with adenoviruses have been reported, where the viral E1A protein sensitizes cancer cells to gemcitabine-induced apoptosis, even without full viral replication. This combination has shown promise in pancreatic and ovarian cancer models, improving tumor cell death and survival in preclinical studies [107].

Reoviruses, such as ReoT3D, have been tested in combination with gemcitabine for treating non-small cell lung cancer (NSCLC). While gemcitabine did not increase viral progeny production like other chemotherapy agents (e.g., paclitaxel or vinblastine), it did modestly enhance PARP cleavage, a marker of apoptosis. However, the synergistic effects of combining reovirus and gemcitabine were only observed in gemcitabine-sensitive NSCLC cells, not in gemcitabine-resistant cell lines. This suggests that the effectiveness of the combination depends on the cancer cells’ responsiveness to gemcitabine [107].

Combination therapy with the oncolytic parvovirus H-1PV and gemcitabine has demonstrated synergistic effects in killing pancreatic cancer cells. Notably, gemcitabine-resistant cancer cell lines were still susceptible to H-1PV infection and oncolysis, suggesting H-1PV as a potential second-line treatment for pancreatic cancers resistant to gemcitabine. In an orthotopic pancreatic cancer model, H-1PV effectively targeted tumors that escaped gemcitabine treatment, extending survival when administered after gemcitabine. However, simultaneous administration of both treatments reduced the therapeutic effect, likely due to negative interactions affecting virus replication. Thus, a sequential, two-step approach is recommended for combining H-1PV and gemcitabine therapy [94].

The synergy between oncolytic herpes simplex viruses (HSVs) and gemcitabine depends on the genetic makeup of the specific HSV strains used. In pancreatic cancer models, two HSV-1 strains, R3616 (with an ICP34.5 deletion) and hrR3 (with an ICP6 deletion), showed enhanced cytotoxicity in vitro when combined with gemcitabine. However, R3616 responded better to the combination, while hrR3 was more sensitive to gemcitabine’s inhibitory effects on viral replication. In a mouse model, R3616 and gemcitabine had a stronger anticancer effect, whereas hrR3 showed antagonism when combined with the drug. Similarly, HSV-1 strain NV1066, with deletions in ICP0, ICP4, and γ134.5, exhibited increased replication and cytotoxicity when paired with gemcitabine. These findings highlight that the success of OV and chemotherapy combinations can vary based on the specific genetic modifications of the viruses [108].

5-Fluorouracil (5-FU): 5-Fluorouracil (5-FU) is a pyrimidine antimetabolite used in chemotherapy, targeting either DNA or RNA depending on its derivatives. Combining 5-FU with oncolytic adenoviruses has shown synergy in cancer treatment, particularly in pancreatic cancer. This synergy is partly due to 5-FU upregulating the Coxsackievirus-adenovirus receptor (CAR), enhancing viral entry. Strategies include improving 5-FU metabolism in cancer cells, delivering proapoptotic molecules, and enhancing 5-FU effects with therapeutic molecules delivered by viruses. Synergy between 5-FU and oncolytic HSV-1 strains, like NV1066 and G207, has also been reported, enhancing viral replication and cytotoxicity while reducing doses for both agents [94,109].

##### DNA Intercalating Agents and OVs

Doxorubicin, an anthracycline, inhibits DNA synthesis by forming a complex with topoisomerase I and II, causing DNA breaks. It has been reported that oncolytic adenovirus ONYX-015 can reverse doxorubicin resistance and synergize with cisplatin, particularly in the presence of wild-type p53. This led to a phase I-II clinical trial combining ONYX-015 with mitomycin C, doxorubicin, and cisplatin, which showed partial responses in advanced sarcoma patients. Doxorubicin also shows additive effects when combined with oncolytic HSV strains (e.g., HSV-1716, G207, and NV1023) in lung cancer and anaplastic thyroid cancer models. Additionally, doxorubicin and VSV combination treatments have demonstrated synergism by inducing degradation of the anti-apoptotic Mcl-1 protein [110].

##### Mitotic Inhibitors

Taxanes (Paclitaxel l): Paclitaxel, an anti-microtubule agent, stabilizes microtubules and prevents their depolymerization, leading to cell cycle arrest. Synergistic interactions between paclitaxel and oncolytic HSVs have been observed, particularly in anaplastic thyroid cancer (ATC) cells. For example, G207, an oncolytic HSV, enhances apoptosis and mitotic arrest in paclitaxel-treated ATC cells. Similar synergism has been reported with HF10 and G47Delta in murine models of colon and prostate cancer, respectively, where paclitaxel enhances viral replication and cytotoxicity. Paclitaxel also improves the oncolytic activity of adenoviruses and reovirus (ReoT3D) by increasing mitotic arrest and apoptosis [94,111,112].

#### 6.1.2. Non-Cytotoxic Targeted Chemotherapy Agents

Various targeted agents, including tyrosine kinase inhibitors, small molecules, and monoclonal antibodies, have shown potential for synergizing with OVs (OVs). These combinations can enhance therapeutic effects by improving immune responses or affecting intracellular pathways. However, it is crucial to ensure that these drugs do not interfere with the OVs’ selective replication in cancer cells, as this could compromise their efficacy [94].

##### Histone Deacetylase Inhibitors (HDIs)

Histone deacetylase inhibitors (HDIs), such as valproic acid (VPA), suberoylanilide hydroxamic acid (SAHA), and trichostatin A (TSA), have gained attention for their ability to suppress the transcriptional activation of interferon-stimulated genes (ISGs). This suppression enhances the effectiveness of OVs by inhibiting innate immune responses in infected tumor cells. For instance, SAHA has been shown to increase the infectivity and replication of vesicular stomatitis virus (VSV) and improve the oncolytic efficacy of other viruses like vaccinia virus (VACV) and herpes simplex virus type 1 (HSV-1) by reducing the IFN response. Additionally, HDIs can enhance the uptake of adenoviruses by upregulating the coxsackievirus-adenovirus receptor (CAR) in cancer cells. However, HDIs’ effects can vary based on the OV strain and the specific HDI used, indicating that further evaluation is necessary. HDIs can also improve the effectiveness of adenovirus-based gene therapies, such as those involving p53 and TRAIL, by enhancing the expression of these therapeutic transgenes and promoting apoptosis [94,113].

##### Epidermal Growth Factor Receptor (EGFR) Inhibitors

Erlotinib, an EGFR signaling pathway inhibitor, binds to the ATP binding site of EGFR, blocking its autophosphorylation and downstream signaling. It is used with gemcitabine for pancreatic cancer. In studies combining erlotinib with oncolytic HSVs G307 and hrR3, the combination showed additive effects in vitro without impacting viral replication. Animal models indicated a modest increase in anticancer effects, but further evaluation of this combination is needed [114].

#### 6.1.3. Arming Oncolytic Viruses to Enhance Chemotherapy

Second-generation armed oncolytic viruses (OVs) designed to express therapeutic transgenes present numerous opportunities to enhance virotherapy as a monotherapy. Additionally, OVs can be engineered with transgenes that sensitize infected cancer cells to specific drugs, thereby improving the effectiveness of drug-based therapies. Many of these strategies utilize gene-directed enzyme prodrug therapy (GDEPT), where viruses selectively deliver enzymes to cancer cells, enabling the conversion of prodrugs into their active, toxic forms. This approach aims to increase the precision of chemotherapy, minimizing the systemic toxicity often associated with conventional treatments. Notably, these systems can also induce a potent bystander effect, further amplifying their overall anticancer activity. However, their success relies on the selective and efficient expression of the drug-activating enzyme within tumor tissues [114].

#### 6.1.4. Oncolytic Viruses and Immune Checkpoint Blockers (Table 3)

##### Anti PD-1 and Anti PD-L1 Antibodies

Immune checkpoint blockade (ICB) is an essential strategy in cancer immunotherapy, targeting inhibitory molecules like PD-1, PD-L1 [115,116], and CTLA-4 to boost antitumor responses. While several FDA-approved antibodies, such as nivolumab and pembrolizumab, have shown success in blocking these pathways, tumors often develop resistance due to mechanisms like PD-L1 downregulation and low CD8^+^ T cell frequency in the tumor microenvironment (TME) [117].

**Table 3:** Combination of oncolytic viruses and immune checkpoint blockers.

Oncolytic Virus	Intervention	Type of Study	The Outcome of Combination Therapy
HSV-1716 [118]	Anti-PD-1	Murine rhabdomyosarcoma model	Increasing CD4^+^ and CD8^+^ T cell-mediated antitumor responses but not Tregs
VVWR/TK-RR-/FCU1 [119]	Immune checkpoint blockers or oxaliplatin	MCA205 fibrosarcoma cells/Mouse model	Inducing abscopal effects on distant untreated tumor cells with defects in type I IFN signaling Inducing immunologic cell death
Vaccinia virus [120]	Anti-PD-L1	Ovarian and colon cancer models	Increasing the infiltration of CD4^+^ and CD8^+^ T cells Promoting the release of perforin, granzyme B, IFN-γ, and the ICOS Reducing the frequency of PD-1^+^CD8^+^ exhausted T cells and virus-induced PD-L1^+^ immunosuppressive cells such as Tregs, TAMs, MDSCs, and DCs
Human reovirus [120]	Anti-PD-1	Glioma model	Improve antitumor responses
VSV-mIFNβ-NIS [121]	Anti-PD-L1	Murine AML cell line C1498 (ATCC TIB-49) AML mouse model	Improving antitumor responses and animal survival compared with Increasing the frequency of monotherapy with anti-PD-L1 or VSV-mIFNβ-NIS Reducing tumor burden in various organs of the studied mice effector CD4^+^ and CD8^+^ TILs in the TME
T-VEC [122]	Anti-PD-1	Patients with stage IIIC to stage IVM1b melanoma	Increasing the number of CD8^+^ T cells, as well as virus-induced PD-L1 expression Suppressing the expression of PD-L1 Improving immunotherapy’s effectiveness through reprogramming the TME
HSV-aPD-1 [123]	Anti-PD-1	MC38 and B16–F10 models	Promoting T-cell infiltration and antitumor immune responses more than unloaded viruses or anti-PD-1 monotherapies Upregulating antigen presentation and IFN pathway-related genes Downregulating the expression of cell adhesion and angiogenesis-associated genes
VT1093M [124]	VT1092M (Express mouse anti-PD-1 antibody)	Murine CT26 colon adenocarcinoma model	Inhibiting primary tumor growth Preventing contralateral untreated tumor growth Generating a vaccine-like immune response, activate tumor-specific T cells Prolonging the OS rate of the studied mice
VACV; JX-594 [125]	Anti-PD-1	EMT6, B16–F10, LLC, MB49, MC38, CT26, and 4T1 cancer cell lines Animal models	Increasing T cell factor-1(TCF-1)^+^ stem-like T Enhancing therapeutic effectiveness. Inducing durable antitumor immunity
ΔTK-Armed-VACV [126]	Expressing anti-PD-1 and anti-4-1BB antibody genes	A549 or 4T1 tumor-bearing mouse model	Induced tumor-specific CD8^+^ T cells Releasing of IFN-γ Inhibiting tumor growth
AdV-D24-ICOSL-CD40L [127]	Anti-PD-1	MUG, Mel-1, and MUG Mel-2 Immunocompetent C57BL/6 melanoma B16V mouse model	Reducing tumor size Increasing the survival rate
V937 [128]	Anti-CTLA4	Open-label, phase II study Unresectable stage IIIC or IV melanoma	Inducing systemic immune activation Durable responses (>26 weeks)
G47Δ [129]	Anti-CTLA4	Murine esophageal SCC cell lines, AKR and HNM007, SCCVII, Hepa1–6, murine melanoma cell line B16–F10, and Vero Animal models	Improving antitumor responses Recruiting of effector T cells into the TME and decreasing the frequency of Tregs at the tumor site Upregulating the expression of various genes involved in inflammatory responses and T cell activation
RFlu-CTLA4 rFlu-huCTLA4 [130]	Expressing anti-CTLA4	H22 mouse model (HCC)	Inhibiting tumor cell growth Prolonging the OS of treated mice Increasing the number of CD4^+^ and CD8^+^ T cells
M1 [131]	Anti-CTLA4	Melanoma and prostatic tumor models	Inducing tumor suppression and lymphocytotoxicity Decreasing Tregs and increasing the infiltration of CD45^+^ immune cells and CD4^+^ or CD8^+^ T cells Inducing the release of IFN-γ, perforin, and granzyme Extending the OS rate compared with monotherapy with M1
G47-mIL-12 [132]	Anti-PD-L1 and anti-CTLA4 (Triple therapy)	GBM model	Triple therapy was more effective than dual therapy using anti-CTLA4 and anti-PD-1 antibodies to improve the survival of animals
Adenovirus [133]	Anti-PD-L1 and anti-CTLA4 (Triple therapy)	TNBC model	Inhibiting tumor growth and prolonged survival Recruiting CD8^+^ T cells, inducing memory T cells Decreasing the number of Tregs and M2 macrophages in the TME
Ad5D24/CCL5/IL-15 [94]	Anti-GD2 CAR-T cell	Neuroblastoma tumor model	Enhancing the infiltration of anti-GD2 CAR-T cells into the TME Eliminating tumor cells
OAd/IL-2/TNF-α [94]	meso-CAR T-cells	BxPC-3, Capan-2, AsPC-1 KrasLSL.G12D/+p53R172H/+ (KPC) mouse pancreatic tumor model	Expressing IL-2 and TNF-α Boosting the functions of mesothelin-redirected CAR-T cells
Adenovirus expressing BiTE targeting EGFR [134]	Anti-FR-α CAR-T cells	SKOV3, HCT116, Panc-1, NCI-H226, C30 Animal models	Promoting CAR-T cells proliferation, activation, and tumor cell killing ability Prolonging the survival of studied mice
CAdVECPDL1 [135]	Anti-Her2 CAR-T cells	Human prostate cancer cell line PC-3, human non-small cell lung carcinoma cell line A549, human HCC cell line HepG2, and human squamous cell carcinoma line SiHa Animal models	Expressing anti-PD-L1 mini-bodies Enhancing antitumor effect of CAR-T cells Prolonging the survival rate of the treated animals with CAdVECPDL1 and anti-Her2 CAR-T cells compared with monotherapy by CAdVECPDL1 or anti-Her2 CAR-T cells
CAd12/PDL1 [136]	CAR-T cells	HNSCC xenograft model	Expressing anti-PD-L1 mini-bodies and IL-12p70 Inducing CAR expression and killing of tumor cells by CAR-T cells
Oncolytic adenovirus (CAd) expressing IL-12p70 [137]	HER2-specific CAR-T cells	The human HNSCC line FaDu and human TNBC cancer cell line MDA-MB468 Animal model	Encoding epitopes to stimulate naïve TCRs Enhancing CAR-T cell proliferation, activity, efficacy, and immune memory

ICBs with other therapies, such as oncolytic virotherapy, present a synergistic opportunity. OVs can increase CD4^+^ and CD8^+^ T cell infiltration into the TME and trigger IFN-γ secretion, complementing the action of ICBs. For example, virotherapy with canerpaturev (C-REV), an attenuated HSV-1 strain, enhances immune responses by increasing activated CD8^+^ TILs, while ICBs inhibit PD-L1 upregulated by the virus. In models such as murine rhabdomyosarcoma, combining anti-PD-1 with OVs like HSV-1716 demonstrated improved CD4^+^ and CD8^+^ T cell responses and stronger antitumor effects compared to either treatment alone [117].

Similarly, engineered vaccinia viruses (VVWR/TK-RR-/FCU1) and human reovirus have shown that combining these viruses with ICBs can shift the balance toward more effective antitumor immunity. This includes greater T cell infiltration, reduction of immunosuppressive cells like Tregs and MDSCs, and enhanced production of cytotoxic molecules such as perforin and granzyme B. Clinical trials, such as those combining T-VEC (an engineered HSV-1) with pembrolizumab, have shown increased CD8^+^ T cells and PD-L1 expression, suggesting improved response when paired with ICBs [138].

The timing of ICB administration in combination therapies is critical. Some studies suggest that using anti-PD-1 alongside OVs shortly after virotherapy maintains the priming of effector T cells while preventing their exhaustion. Moreover, genetic manipulation of OVs to express ICB antibodies or cytokines, such as TNF-α or IL-12, holds promise, although this approach has not always proven superior to combination therapies using separate administration of OVs and ICBs.

Taken together, the combination of oncolytic virotherapy and immune checkpoint inhibitors offers a powerful method to enhance immune responses, reprogram the TME, and overcome resistance mechanisms in cancer therapy [139,140].

##### Anti-CTLA4 Antibodies

Ipilimumab, a CTLA4-blocking antibody, was FDA-approved in 2011 to treat melanoma but can cause immune-related adverse effects. To improve outcomes, combining ipilimumab with OVs has shown promise. Clinical trials, such as those using T-VEC with ipilimumab, have demonstrated effective tumor inhibition in melanoma patients with minimal adverse effects. Similarly, combining oncolytic coxsackievirus A21 (V937) with ipilimumab led to durable immune responses and manageable toxicity.

Other OVs, like engineered HSV-1 and influenza A viruses, have shown enhanced antitumor responses when combined with ipilimumab, primarily by increasing immune cell infiltration (CD4^+^, CD8^+^ T cells) and reducing immunosuppressive Tregs in the tumor microenvironment. These combinations result in higher production of key antitumor cytokines (e.g., IFN-γ) and improved tumor control.

Overall, combining OVs with CTLA4 inhibitors enhances immune-mediated tumor destruction, offering a promising future for cancer treatment [141,142].

#### 6.1.5. Oncolytic Viruses and CAR-T Cell Therapy

Combining genetically modified OVs with CAR-T cells enhances their infiltration and activity in solid tumors. OVs engineered to express cytokines like IL-15 or immune checkpoint inhibitors such as anti-PD-L1 mini-antibodies boost CAR-T cell effectiveness. These combinations improve tumor targeting, proliferation, and survival outcomes, overcoming some resistance mechanisms. However, high-dose treatments risk off-target toxicity, which can be mitigated by miRNA-targeting systems. Overall, OVs enhance CAR-T cell therapy by increasing immune cell recruitment and antitumor activity [142].

#### 6.1.6. Oncolytic Viruses and Cyclooxygenase 2 (COX-2) Inhibitors

COX-2 inhibitors, a class of nonsteroidal anti-inflammatory drugs, can promote tumorigenesis by increasing prostaglandin E2 and enhancing angiogenesis through VEGF expression. COX-2 is also expressed in B lymphocytes, and inhibitors reduce antibody production. In an ovarian cancer model, combining the COX-2 inhibitor Celecoxib with oncolytic vaccinia virus (VACV) demonstrated synergistic anticancer effects. Celecoxib’s immunosuppressive action reduced neutralizing antibodies against VACV, enabling a second virus dose without compromising macrophage and CD8^+^ T cell infiltration, though CD4^+^ T cells were slightly reduced. These effects are likely due to immunosuppression rather than increased virus replication [143].

## 7. Combination of Oncolytic Viruses and Radiotherapy (Table 4)

Radiation interacts with various viruses to enhance their effects. For example, the Tax protein in HTLV-1 and Tat in HIV-1 inhibit DNA repair and promote radiosensitivity, while the Epstein–Barr virus inhibits p53 and DNA repair. OVs such as reovirus, adenovirus, and HSV-1 can also interact with the DNA damage response (DDR) pathway to radiosensitize cells. Reovirus downregulates DNA repair genes, while adenovirus activates the NHEJ pathway and degrades the MRN complex, enhancing radiosensitivity. HSV-1 degrades DNA-PKcs, inhibiting DNA repair and increasing viral replication in glioblastoma cells [96,144].

**Table 4:** Clinical studies on oncolytic viruses and radiation therapy.

Study Design	Endpoint(s)	Patients/Subjects	Intervention	Outcome
Phase 2 trial [96,145]	To assess the possibility and apoptosis induction mechanisms after gene transfer of adenoviral/Ad-p53 (INGN 201) and RT.	19 patients with nonmetastatic NSCLC are not eligible for chemoradiation/surgery.	Ad-p53 (INGN 201) was injected intratumor on three different days (Days 1, 18, and 32) in an outpatient setting; RT (30 2 Gy) was delivered concomitantly over 6 weeks to the primary tumor and mediastinal lymph nodes.	Common adverse events were grade 1/2 fevers and chills (79 and 53%). 3-month follow-up biopsies: no viable tumor (63% patients), viable tumor (16% patients), and the rest not assessed.
Phase 1 trial [96]	To establish the DLT after the administration of OV concurrent with conventional-dose 3D-CRT and increasing duration of 5-FC + vGCV prodrug therapy. Moreover, transgene expression and assessment of therapeutic response are demonstrated by prostate biopsy and serum PSA level.	15 patients with prostate cancer with adenocarcinoma subtype (stage T1c to T4).	A single intraprostatic injection of replication-capable Ad5-CD/TKrep adenovirus particles, followed by valganciclovir prodrug therapy and 5-fluorocytosine for 1 (Cohorts 1–3), 2 (Cohort 4), or 3 (Cohort 5) weeks along with 70–74 Gy 3D-CRT, 2 Gy per fraction.	There were no DLT and intervention-related adverse reactions. Transgene expression persisted in the prostate for up to 3 weeks after the OV injection. The average PSA half-life in subjects who received the prodrug therapy for more than 1 week was significantly shorter than 1 week (0.6 vs. 2.0 months; *p* < 0.02), faster than the previous report for subjects who were given 3D-CRT conventional-dose only (2.4 months). In the intermediate-risk group, 0/12 patients were positive at 1-year followed up prostate biopsy.
Phase 1 trial [146]	Evaluation of safety, tolerability, feasibility, and antitumor activity of intratumoral injection of TNFerade (adenovector) in parallel with RT.	36 cancer patients (pancreatic, NSCLC, breast, colorectal, head and neck, esophagus, bladder, sarcoma biliary, primary liver, melanoma, and adrenal).	TNFerade was given via intratumoral injection twice weekly (Weeks 1–2), then once weekly (Weeks 3–6). 1.8–2.0 Gy/day of radiation was given until a total dose of 20–66.6 Gy.	The most common toxicities included fever, injection site pain, and chills (22%, 19%, and 19%, respectively). No grade 3–4 toxicities were observed. 70% of 30 patients demonstrated CR, PR, or MR at 4 weeks post-treatment.
Phase 1 trial [147]	To evaluate the safety of concomitant TNFerade and radiotherapy.	14 patients with soft tissue sarcoma of the limb (liposarcoma, leiomyosarcoma, chondrosarcoma, and spindle cell sarcoma malignant fibrous histiocytoma).	3 escalating dose levels of TNFerade were given via intratumoral injections, twice a week (Week 1) then once a week (Week 2–5) throughout single daily fraction of radiation therapy All patients received daily concomitant RT in 1.8–2.0 Gy per fraction for a total dose of 36–50.4 Gy.	No DLT was observed. The most common adverse events were grade 1–2 chills, fever, fatigue, and flu-like symptoms (50%, 43%, 36%, and 21%, respectively); 85% of subjects showed an objective tumor response with 2 CR, 9 PR, and 1 SD.
Phase 3 RCT [148]	Safety and efficacy of adenovirus with the cloning of HSV-tk (AdvHSV-tk) and GCV administration via intravenous injection.	36 subjects (operable primary or recurring malignant glioma); AdvHSV-tk gene therapy was given to 17 subjects and control treatment (surgery with/without radiotherapy) was given to 19 subjects	AdvHSV-tk was injected directly into the healthy tissue of the wound after tumor resection in the intervention group. Subsequently, GCV was given intravenously after the gene therapy. In all groups, steroids, antiepileptics, and post-operative 60 Gy RT were given to the subjects.	The intervention group had a 65% longer median survival than the control (62.4 vs. 37.7 weeks, *p* = 0.0095). AdvHSV-tk intervention was well-tolerated without the occurrence of important safety issues.
Phase 1 trial [96]	To establish the toxicity-related intraprostatic injection of OV + vGCV prodrug therapy, 5-FC, and IMRT. Another endpoint is to determine the presence of positive prostate needle biopsies at 6, 12, and 24 months and post-treatment PSA	9 subjects (5 intermediate-risk and 4 high-risk prostate cancer)	A single intraprostatic injection of Ad5-yCD/mutTKSR39rep-ADP, then the subjects received 2.6 weeks (13 days, only on weekdays) of 5-FC + vGCV prodrug therapy + 74 Gy IMRT. In another cohort, two intraprostatic injections of OV were administered, followed by a subsequent 2.6 weeks of 5-FC + vGCV prodrug therapy + 74 Gy IMRT.	The investigational therapy found low toxicity (grade 1–2); there were no DLT and treatment-related serious adverse events. 7 of 8 patients were negative in the last post-treatment prostate biopsies (6 or 12 months later).
Phase 1/II trial [96]	Safety and recommended schedule/dose of JS1/34.5-/47-/GM-CSF (HSV encoding GM-CSF) for future study. Antitumor responses (pathologic, radiologic, recurrence rates, and survival) were also monitored	17 head and neck cancer subjects [oropharynx (palatine tonsil and base of tongue), supraglottis, and hypopharynx (pyriform fossa)]	JS1/34.5-/47-/GM-CSF was injected into malignant lymph node(s) on the cervical region using a scheduled and escalated dosing manner (no injections were done to mucosal disease) All patients received radiotherapy (70 Gy in 35 daily fractions) and standard-dose cisplatin (100 mg/m^2^ body surface area) chemotherapy	All subjects had at least one OV-related adverse event, 86% grade 1–2, one grade 3–4 observed in each subject. 82.3% of 17 patients showed tumor response by Response Evaluation Criteria in Solid Tumors, and 93% of neck dissected patients showed pathologic complete remission. HSV replication was found with a titer higher than the input dose. All subjects were seropositive at the end of the intervention. No locoregional reappearance; disease-specific survival was 82.4%, median follow-up: 29 months (19–40 months)
Phase 1 trial [96]	To determine safety, feasibility, antitumor activity, and viral replication of combining intratumoral administration of reovirus type 3 Dearing (RT3D) and radiation to subjects with advanced malignancy during fractionated radiotherapy. This study will give recommendations on the dose combination schedule	25 patients with squamous cell carcinoma of the skin, melanoma, lung, head and neck cancer, pancreas, ovarian, esophagus, colorectal, and unknown primary.	At the first stage (phase 1a): local tumor radiation to a dose of 20 Gy in 5 successive daily fractions + 2 intratumoral injections of RT3D in consecutive log dose-escalating for the cohorts consisting of 3 patients. In the second stage (phase 1b), local tumor radiation to a dose of 36 Gy (12 fractions) over 16 days in parallel with 2, 4, or 6 intratumoral RT3D doses.	No DLT was observed. No viral shedding was found on RT-PCR of blood, urine, stool, and sputum. Most of the common toxicities were grade 1–2, such as pyrexia, flu-like symptoms, vomiting, asymptomatic lymphopenia, and neutropenia. In the low-dose (5 4 Gy) radiation group, out of 7 patients, 5 had SD and 2 PR. Out of 7 patients in the high-dose (12 3 Gy) radiation group, 2 had SD and 5 had PR.
Phase 2/3 trial [96]	To determine the safety and efficacy of OAMCGT + IMRT for intermediate-risk prostate cancer. Other aims were prostate biopsy positivity at 2 years, quality of life (QOL), no apparent biochemical/clinical failure, metastases, and survival.	44 subjects who had intermediate-risk prostate cancer. (OAMCGT + IMRT: 21 patients; IMRT: 23 patients).	The intervention group received an Ad5-yCD/mutTKSR39rep-ADP adenovirus via a single intraprostatic injection. Then, subjects in the same group were given 5-FC and vGCV orally for 2 weeks (given only during weekdays). All patients received 40 × 2 Gy of IMRT.	Significant differences in gastrointestinal or genitourinary events and quality of life among the two groups were not observed. However, neutropenia, low-grade influenza-like symptoms, transaminitis, and thrombocytopenia were more prevalent in the intervention group. Subjects in the intervention group showed 42% less biopsy positivity at 2 years compared with the other group. Biopsy positive in the intervention vs. control group: 33% vs. 58%.
Phase 1 trial [96]	To know the safety of direct inoculation of HSV/G207 into recurrent malignant gliomas followed by focal radiation to the tumor. Other outcomes were efficacy, overall survival, and radiographic and performance response HSV/G207-degree of post-treatment viral shedding and specific antibody response.	9 GBM subjects with progressive disease despite surgery (resection or stereotactic biopsy), chemotherapy, and radiation therapy.	G207 was inoculated using stereotactic technique into 5 enhanced parts of the tumor edge for 2 min to avoid any reflux. All subjects received 5 Gy radiation therapy.	No subjects experienced serious adverse effects due to the intervention and no subjects showed any evidence of HSV encephalitis. The most common adverse events were headache, seizure, hemiparesis, and fever. 6 subjects had SD or PR. Quantitative PCR of HSV in blood serum stayed negative in all subjects. The median survival was 7.5 months (time from G207 inoculation to death).
Phase 1 trial [149]	To determine the safety of GL-ONC1 when delivered intravenously with chemoradiation to patients with primary, non-metastatic head and neck cancer.	19 patients with stage IV head and neck carcinoma.	Patients received GL-ONC1 intravenously with concurrent chemotherapy (cisplatin 100 mg/m^2^ on Days 1, 22, and 43) and radiation. Patients in Cohort 1 received 3 × 10^8^ pfu on Day 3; Cohort 2, 1 × 10^9^ pfu; Cohort 3, 3 × 10^9^ pfu; Cohort 4, 3 × 10^9^ pfu on Days 3 and 8; Cohort 5, 3 × 10^9^ pfu on Days 3, 8, 15, and 22.	Follow-up was done at the median of 30 months, which resulted in 7 treatment failures (3 locoregional, 3 distant, and 1 both local and distant) and 7 deaths (5 due to progression of head and neck carcinoma, 1 due to a second primary cancer of gastrointestinal origin, and 1 due to non-cancer cause). Post-treatment PET/CT at 4 months in 18 patients showed negative in 11 patients, partial response in 4 patients, positive in 3 patients, and death at 3 months post-treatment in 1 patient. PFS at 1 and 2 years was 74.4% and 64.1%, respectively.

Radiation combined with OV enhances viral oncolysis through four mechanisms: increased viral uptake, gene expression, transgene activation under radio-inducible promoters, and viral replication. Radiation-induced viral gene expression has been observed in studies on measles and adenovirus, which can augment therapeutic efficacy. For example, a combination of adenovirus encoding TNF-α and radiation significantly increased TNF-α levels. While enhanced viral replication is cell-line dependent, synergy between OV and radiation can still occur even without viral replication, as shown with HSV in glioblastoma models [150,151,152,153].

## 8. Systemic Delivery of Oncolytic Virus

Systemic delivery of OVs is a cutting-edge approach to the treatment of head and neck cancer and aims to distribute OVs throughout the body to target both primary tumors and distant metastases. This method is particularly beneficial for the treatment of undiagnosed metastatic advanced disease and in cases where the disease is inaccessible, as OVs can activate systemic tumor immunity [10,30,111,154,155]. However, to achieve efficient systemic delivery, several biological barriers must be overcome, such as the immune response, which can neutralize the virus before it reaches the tumor site, and the vascular and interstitial pressure within tumors, which can hinder virus penetration. Initially, systemic administration was associated with severe flu-like symptoms, yet its toxicity profile remained favorable compared to conventional cancer therapies [65,156]. Increased interstitial hydrostatic pressure due to fibrosis and vascular abnormalities presented another physical barrier to successful virus transmission. Viruses could escape by flowing back into the circulation or through the injection site, resulting in reduced efficacy and increased systemic toxicities [157]. Serum neutralization and hepatotoxicity are key challenges in developing systemic delivery. A neutralizing antibody response has been reported in almost every patient treated with the virus, but this has not been associated with a therapeutic response or lack thereof [158]. Therefore, systemic administration of OVs must overcome poor target distribution, existing specific antiviral immunity, and innate immune responses [159]. Recent advances have focused on improving the systemic delivery of OVs through various strategies. One approach is to modify the virus surface to evade immune recognition. For example, coating OVs with polyethylene glycol (PEG) has been shown to increase their circulation time by reducing recognition and clearance by the immune system [37,160]. Another promising strategy is the use of cell carriers such as mesenchymal stem cells (MSCs), which can harbor tumors and release the virus locally [161,162,163]. The advantage of using stem cells is that they allow high levels of homing even at a low infectious dose and provide a protective environment for the virus, protecting it from the immune system during transport [164]. In addition, MSCs are suitable for loading with various chemotherapeutic agents and oncolytic viruses due to their hypoimmune nature, inherent tumor tropism, ease of isolation, and potential for differentiation across multiple lineages. These intrinsic features, combined with the possibility of genetic manipulation, make MSCs versatile tumor delivery vehicles that can be used for selective tumor delivery of various chemotherapeutic and biologic therapeutics in vivo. MSCs can act as biofactories for the local production of chemical or biological anticancer agents at the tumor site. MSC-mediated immunotherapy could facilitate the sustained release of immunotherapeutics targeted to the tumor site and achieve therapeutic concentrations without the need for repeated systemic administration of high doses [165]. Furthermore, preclinical studies have shown the potential of combining OVs with immune checkpoint inhibitors to improve therapeutic efficacy. This combination may help modulate the tumor microenvironment, thereby favoring viral replication and spread [166]. Nanotechnology-based active and passive drug delivery systems are also being explored to improve the targeting and stability of OVs in circulation [167,168].

## 9. Safety of Oncolytic Virotherapy

Safety remains a crucial aspect in the clinical use of oncolytic virotherapy, particularly in head and neck cancer. The therapeutic use of OVs requires strict safety protocols to ensure that the virus selectively targets cancer cells while sparing normal tissue. Additionally, concerns about viral shedding and transmission to healthy individuals must be carefully addressed. The interactions of replicating viruses with the host and the environment are difficult to predict and require precautions to minimize exposure to healthcare providers, family members, and other patient contacts. Theoretically, non-pathogenic viruses can acquire new pathogenic properties, or modified viruses can revert to their wild type. These safety concerns continue to influence the development of oncolytic virus therapy [169,170]. Furthermore, although gene deletions are necessary for tumor selectivity, some mutations may result in reduced antitumor efficacy due to inefficient entry, replication, or lysis of tumor cells. It remains a challenge to genetically modify the viruses to ensure safety while increasing effectiveness. Other hurdles include maintaining efficient virus spread and avoiding the host immune system [171]. The continuous development of virology and genetic engineering technologies has allowed researchers to genetically edit OVs, significantly improving their safety, specificity, and effectiveness in cancer treatment [21]. Digital techniques, navigation surgery, and artificial intelligence have been integrated into the overall treatment of head and neck cancer [172], further improving the precision, safety and effectiveness of treatment plans [173,174,175]. Since OVs have multiple mechanisms of action, ensuring safety through clinical validation with statistically proven clinical trials is crucial [176,177]. Current literature has confirmed the safety profile of OVs and demonstrated that they can be administered with minimal side effects. Common side effects include mild flu-like symptoms, fever, and local inflammation, which are generally manageable [178]. To increase safety, OVs are often genetically engineered to delete or attenuate virulence genes, thereby reducing their pathogenicity in normal cells [38,179]. Compared to traditional cancer treatments, modern OVs rely less on the expression of specific receptors and are less susceptible to mutations or transcriptional resistance. They exhibit high safety and specificity while avoiding drug resistance issues that may occur during chemotherapy [69,111]. OVs possess multiple antitumor mechanisms and have broad application potential, often synergizing with traditional cancer therapies [180]. The combination of standardized chemotherapy with OVs enhances the antitumor effect, ensures safety, and prolongs patient survival [181]. Recent research is focused on developing advanced monitoring techniques to detect and control viral shedding. These include real-time PCR methods to track viral genomes in patient samples to ensure that potential spread of the virus can be quickly detected and contained [182]. Furthermore, improvements in viral vector design, such as the incorporation of microRNA targeting sequences, have further increased the specificity and safety of OVs by restricting their replication to cancer cells [88] Some OVs have been abandoned for cancer therapy due to ineffectiveness, intolerable toxicity, and serious safety concerns [183]. In many Ovs used for human trials, few adverse events and limited deaths have been associated with viral replication [184]. Compared to the toxicity of standard cytotoxic drugs, the toxicity of Ovs in viroimmunotherapy is satisfactory. Overall, off-target effects and viral mutation/transmission remain the hallmarks of their safety problems [158]. The interactions of these engineered oncolytic replicating viruses with the host and the environment are difficult to predict. Therefore, Ovs must be structured to have enough virulence to significantly reduce tumor mass while avoiding side effects in patients [31]. Despite special consideration of engineered Ovs and the acceptance of newer oncolytic viruses with low seroprevalence and the ability to avoid neutralizing antibodies, there is an increased risk of virus spread with less favorable safety profiles that have been documented for many genetically engineered Ovs evaluated [185]. As the field advances, ongoing clinical trials and post-marketing surveillance will be critical to continually evaluate and improve the safety of oncolytic virotherapy. Ensuring robust safety measures and comprehensive patient monitoring will be key to wider acceptance and acceptance of this innovative cancer treatment.

## 10. Challenges and Future Directions

Oncolytic virotherapy presents a promising treatment strategy for HNSCC by utilizing genetically modified viruses that selectively infect and lyse cancer cells. Despite its potential, several challenges hinder its widespread application. One major challenge is efficient delivery and distribution within the tumor [186]. The dense extracellular matrix and abnormal vasculature typical of HNSCC can impede the virus from penetrating deeply into the tumor tissue [186,187]. Additionally, the host immune system can rapidly neutralize the virus, especially in patients with pre-existing immunity to the viral vectors used. Ensuring the virus specifically targets cancer cells without affecting healthy tissues is critical, as any off-target effects could lead to significant toxicity. Furthermore, maintaining the genetic stability of the engineered viruses is essential to prevent them from mutating into a pathogenic form [187].

## 11. Challenges in Penetration

In carcinomas, the intracellular junctions of epithelial cells act as barriers to the penetration of high molecular weight therapeutic agents, contributing to resistance. During metastasis, epithelial-to-mesenchymal transition (EMT) and mesenchymal-to-epithelial transition (MET) tighten these junctions, complicating treatment. Specifically, epithelial junctions hinder the penetration of OVs such as adenoviruses. Some adenoviruses, like HAdV-B3, can overcome these barriers by releasing penton-dodecahedra (Pt-Dd) early in infection, whereas commonly used HAdV-C5 does not [73,188]. Modifications to Ad5, such as expressing an epithelial junction opener, enhance its anti-tumor effect by increasing its affinity to desmoglein 2 (DSG2), a protein over-expressed in epithelial cancers, thereby improving virus penetration [73,188].

The extracellular matrix (ECM) also presents a significant obstacle to the dispersion of anticancer agents within tumors. Strategies to enhance OV penetration include pretreatment with collagenase or co-administration with hyaluronidase, which degrade components of the ECM, improving virus spread. Engineering OVs to express matrix metalloproteinases further aids in degrading tumor-associated glycosaminoglycans, enhancing diffusion and therapeutic efficacy. In addition to ECM degradation, inducing cancer cell apoptosis can facilitate viral spread. Apoptosis caused by cytotoxic agents and caspase-8 activation creates channel-like structures and void spaces, improving the intratumoural penetration and anti-tumor efficacy of oncolytic viruses, as demonstrated with oncolytic HSV [189,190].

## 12. Tumor Cell Targeting

Despite the therapeutic benefits of direct T-VEC administration for melanoma, systemic delivery of OVs often fails due to insufficient tumor cell targeting and transduction. To address this, surface modifications have been employed to enhance tumor cell specificity. For instance, the initial interaction between HAdV-C5 fiber protein and CAR (Coxsackie-Adenovirus Receptor) on target cells is followed by the RGD (arginine-glycine-aspartic acid) motif’s interaction with host cell integrins, triggering clathrin-mediated endocytosis. Since CAR expression is often down-regulated in tumors, modifications like inserting an RGD motif into the adenovirus fiber knob domain have significantly improved infection efficiency and anti-tumor activity in CAR-negative tumors [191,192,193].

Another strategy involves using different adenovirus serotypes with natural affinities for cancer cells. For example, HAdV-G52 binds to polysialic acid, highly expressed in cancers like lung and brain. However, modifications are needed to mitigate potential neurotropism. Additionally, targeting can be enhanced through antibody-based methods, such as fusing single-chain variable fragments (scFvs) with capsid proteins or creating fiber chimeras, to redirect adenoviruses to cancer cells [194].

Bispecific adapters provide another innovative targeting approach. These adapters have two arms: one binds to the virus and the other to tumor cells. For example, a bispecific adapter with a polySia-binding scFv domain and CAR ectodomain effectively targets lung cancer cells overexpressing polysialic acid, improving survival in tumor-bearing mice. Another adapter fusing CAR ectodomain with CXCL12 enhances infectivity in CXCR4-positive cancer cells while reducing hepatotoxicity. Similarly, an adapter combining EGFR-binding scFv with the N-terminal domain of nectin-1 has been developed to redirect HSV-1 to EGFR-expressing tumors [195,196].

## 13. Pre-Existing or Previous Immunity

Preexisting immunity from previous infections or vaccinations poses a challenge for OV therapy, as it can lead to a short half-life following intravenous delivery. “Stealthing” techniques, such as coating oncolytic adenoviruses with polymers like N-(2-hydroxypropyl) methacrylamide (HPMA), polyethylene glycol, and polyamidoamine, help protect the viruses and extend their half-life. These polymers can be conjugated with tumor-targeting moieties (e.g., peptides, antibodies) to increase selectivity [197]. However, non-degradable polymers can interfere with viral binding, so degradable polymers that respond to tumor microenvironment conditions (e.g., hypoxia, low pH) are being explored, although progeny virions won’t inherit these modifications. Cellular carriers, including immune cells, transformed cells, and progenitor cells, offer another method to protect OVs from neutralizing antibodies and reduce toxicity. Stem cells, in particular, are promising due to their natural tumor-homing abilities, attracted by tumor microenvironment factors like hypoxia and angiogenesis signals. For example, neural stem cells (NSCs) loaded with oncolytic adenoviruses show enhanced tumor targeting. Using autologous stem cells is preferable to avoid immune rejection, though their quality and quantity may vary in patients who have undergone extensive treatments [163].

Antiviral cytokines, like various types of interferons (IFNs), can impede the efficacy of OVs by inhibiting viral replication. Histone deacetylase (HDAC) inhibitors, such as valproic acid (VPA), can mitigate these antiviral responses by inducing epigenetic changes. Pretreatment with VPA has been shown to enhance oncolytic virus replication and reduce immune cell infiltration in the tumor microenvironment. Additionally, inhibitors like sunitinib and bevacizumab enhance OV efficacy by targeting antiviral enzymes and reducing interstitial fluid pressure, respectively, promoting better viral diffusion and replication within tumors. Combining OVs with anti-angiogenic agents or immunosuppressive chemotherapies has shown synergistic effects, enhancing both tumor targeting and viral spread [198,199].

## 14. Hypoxic Environment

Hypoxia in solid tumors affects OVs in various ways. It reduces the replication and lytic potential of adenoviruses but does not impact surface receptor expression. Hypoxia can induce cell cycle arrest, affecting viruses like adenoviruses that depend on cell cycle progression for replication [56]. Alcoceba et al. [200] developed an oncolytic adenovirus with an E1A gene controlled by a hypoxia-responsive promoter, enabling it to replicate under hypoxic conditions. Studies by Aghi et al. [201] and Fasullo et al. [202] showed that hypoxia enhances the replication of oncolytic HSVs, possibly due to HSV’s natural preference for low-oxygen cells or oxygen-derived free radical-induced DNA damage. Hypoxia-inducible factor-1α (HIF-1α) also activates genes involved in HSV replication. Infection with oncolytic NDV AF2240 degrades HIF-1α under hypoxia, down-regulating HIF-1α target genes in cancer cells. Other viruses, such as the vesicular stomatitis virus and the vaccinia virus, also replicate better under hypoxic conditions [203].

## 15. Patient Factors

The key question remains: which patients are suitable for oncolytic virotherapy? Currently, no reliable predictive biomarkers exist to identify patients likely to respond to this treatment. Most patients receiving oncolytic virotherapy have already undergone multiple cycles of conventional cancer treatments, leading to a compromised immune system and significantly altered tumors. Zloza et al. [204] conducted a study measuring changes in peripheral blood mononuclear cell gene expression before and after treatment with oncolytic vaccinia virus in melanoma patients. Microarray data showed that 301 genes were up-regulated and 960 were down-regulated post-treatment. This study suggested that immunoglobulin-like transcript 2 could serve as a therapeutic biomarker for oncolytic vaccinia virus treatment. Another study identified high-mobility group box 1 as a potential predictive and prognostic biomarker for oncolytic adenovirus treatment [52].

## 16. Conclusions

In conclusion, future oncolytic virotherapy aims to overcome delivery and selectivity challenges through advanced systems and virus engineering. One approach uses nanoparticles or protective carriers to shield the virus from the immune system and target tumors. Targeting moieties like antibodies or ligands can improve delivery specificity. Genetic modifications can enable viruses to express therapeutic genes that induce apoptosis or stimulate an anti-tumor immune response. Combining different Ovs with complementary actions may also overcome tumor resistance and enhance efficacy. Integrating oncolytic virotherapy with immunotherapies and personalized medicine is crucial for maximizing potential. Combining viruses with immune checkpoint inhibitors or other immunotherapies can enhance the immune response against tumors, addressing the immunosuppressive microenvironment in HNSCC. Engineering viruses to produce cytokines that activate local immune cells can further potentiate this effect. Personalized approaches, such as identifying biomarkers to predict patient response and designing tailored viruses, can improve outcomes. Rigorous clinical trials are needed to ensure safety and efficacy, while regulatory approvals must meet high standards. Continued research, testing, and innovation are essential to establish oncolytic virotherapy as an effective treatment for HNSCC.

## Figures and Tables

**Table 1:** Clinical trials for HNSCC oncolytic virotherapies.

Clinical trials.gov ID	Sponsor	Location	Phase	Virus Description	Intervention	Original Enrollment	Actual Enrollment	Recruitment Status	Publication Status
NCT00832559	Viralytics	Australia	1	Coxsackievirus A21 is a naturally occurring common cold virus that preferentially infect and kill cancer cells expressing the receptors ICAM-1 and/or DAF. This virus is known to cause self-limiting upper respiratory infections and has been used previously to challenge therapies against the common cold. The virus is not generically modified	CAVATAK	9	4	Terminated	NA
NCT01017185	Takara Bio Inc.	USA	1	HF10 is a naturally occurring, non-genetically modified herpesvirus that has demonstrated selective oncolytic properties. While HF10 is known to cause mild, self-limiting skin lesions or flu-like symptoms in healthy individuals, its therapeutic potential lies in its ability to selectively replicate within and destroy tumor cells, sparing normal, healthy tissue	HF-10	18	28	Completed	Not available
NCT01584284	Genelux Corporation	USA	1	GLO1C1 belongs to the Herpesviridae family, specifically a modified strain of the herpes simplex virus (HSV). In particular, GLO1C1 targets tumor cells by exploiting their immune evasion mechanisms, often associated with certain cell surface proteins or altered immune pathways.	GL-ONC1	24	19	Completed	Not available
NCT01846091	Mayo clinic	USA	1	MV-NIS (measles virus-nuclear iodide symporter) is based on the measles virus. It has been genetically modified to express the nuclear iodide symporter (NIS), a protein that allows the virus to be tracked using imaging techniques and facilitates the uptake of radioactive iodine into infected cells, making it useful both as a therapeutic and a diagnostic tool.	MV-NIS	18	12	Completed	Not available
NCT05859074	Memorial Sloan Kettering Cancer Center	USA	1	MQ-710 belongs to the herpesvirus family and works by selectively infecting and replicating within tumor cells, leading to their destruction while activating the immune system to target additional cancer cells	MQ710	56	56	Recruiting	NA
NCT02626000	Amgen	Australia, Austria, Belgium, Canada, France, Greece, Italy, Spain, Switzerland, UK, USA	1b/3	Talimogene laherparepvec (T-VEC), an oncolytic herpes simplex virus engineered to selectively replicate within tumors. T-VEC works by lysing tumor cells and releasing granulocyte-macrophage colony-stimulating factor, which enhances the immune response, potentially improving pembrolizumab’s efficacy in stimulating T-cell activity against the tumor	Talimogene Laherparepvec With Pembrolizumab	40	36	Completed	Published
NCT01161498	BioVex Limited	UK, USA	1	Talimogene laherparepvec (T-VEC) belongs to the Herpesviridae family, specifically a modified strain of herpes simplex virus type 1 (HSV-1). The Herpesviridae family is known for its ability to establish latency in host cells, but in the case of T-VEC, it has been genetically engineered to preferentially infect and lyse tumor cells without causing latency.	Talimogene Laherparepvec + Radiation/Cisplatin	528	5	Terminated	NA
NCT05743270	Replimune Inc.	Czechia, France, Germany, Greece, Poland, Spain, UK, USA	2	RP3 leverages a modified herpes simplex virus (HSV-1) to target and kill cancer cells. RP3 has been genetically engineered to carry additional immunostimulatory genes, including a modified protein from the gibbon ape leukemia virus (GALV) that enhances the killing of tumor cells and the expression of immune activators like anti–CTLA-4, CD40L, and 4-1BBL, all of which promote an anti-tumor immune response.	RP3 in combination with carboplatin, paclitaxel and then nivolumab	130	0	Withdrawn	NA
NCT00625456	Jennerex Biotherapeutics	Canada, USA	1	The recombinant vaccinia virus armed with GM-CSF (granulocyte-macrophage colony-stimulating factor) is an oncolytic virus engineered to both kill cancer cells. The vaccinia virus, commonly used for smallpox vaccines, is adapted for oncolytic therapy by deleting genes that restrict its replication to tumor cells.	Recombinant Vaccinia GM-CSF (JX-594)	24	23	Completed	Not available
NCT03740256	Baylor College of Medicine	USA	1	CAdVEC belongs to the Adenoviridae family, which includes viruses that can infect various cell types in humans and animals. Adenoviruses are non-enveloped, double-stranded DNA viruses known for their ability to deliver genetic material into cells, making them suitable for use in gene therapy and oncolytic virotherapy.	CAdVEC	39	45	Recruiting	NA
NCT05222932	TILT Biotherapeutics Ltd.	Finland, USA	1	TILT-123 selectively targets cancer cells while activating immune responses. It belongs to the adenovirus family, modified to deliver immune-stimulating cytokines, specifically interleukin-2 (IL-2) and tumor necrosis factor-alpha (TNF-α), directly into the tumor microenvironment.	TILT-123 and avelumab	15	15	Recruiting	NA
NCT04348916	Oncorus, Inc.	Canada, USA	1	ONCR-177 is an engineered oncolytic herpes simplex virus type 1 (HSV-1) designed to treat solid tumors through intratumoral administration. It has been modified to express multiple immune-stimulatory transgenes, including IL-12, FLT3LG, CCL4, and antibodies targeting PD-1 and CTLA-4, which help stimulate a potent anti-tumor immune response.	ONCR-177	77	66	Terminated	NA
NCT04685499	Weill Medical College of Cornell University	USA	2	OBP-301, also known as Telomelysin^®^, is an oncolytic adenovirus derived from human adenovirus type 5. It is genetically engineered to selectively replicate in cancer cells, facilitated by the incorporation of the human telomerase reverse transcriptase (hTERT) promoter.	OBP-301	36	1	Terminated	NA

**Table 2:** Examples of Chemotherapy and OV Combination Treatments with Known Mechanisms of Synergy [95].

Chemotherapy Agent	OVs	Primary Mechanism of Synergy with OVs
Cyclophosphamide	Herpesviruses Reovirus Ade- noviruses Poxviruses Pa- ramyxoviruses	Suppression of the host’s innate and adaptive anti-viral immune responses leads to increased and prolonged viral replication [95,98]
Cisplatin	Herpesviruses with γ_1_34.5 deletions	Upregulation of GADD34 improves viral replication [95,99]
Gemcitabine	Adenoviruses	E1A expression counteracts drug resistance mechanisms [100]
5-FU	Adenoviruses Herpesviruses	Upregulation of CAR Upregulation of cellular RR [101]
Doxorubicin	VSV	Viral degradation of Mcl-1 enhances drug sensitivity [99]
Taxenes	Herpesviruses with γ_1_34.5 deletions Adenoviruses	Virus replication is enhanced in drug resistant cells with an upregulated Raf/MEK/ERK pathway E1A expression increases drug sensitivity and anti-tumor immune responses [95]
Histone deacetylase inhibitors	Rhabdoviruses Herpesviruses Poxviruses Adenoviruses	Inhibition of IFN production in infected cancer cells leads to enhanced viral replication Upregulation of CAR and Upregulation of virally delivered transgenes [102]
Rapamycin	Rhabdoviruses Poxviruses Poxviruses (MYXV) Adenoviruses	Impaired IFN production in infected cancer cells enhances viral replication Upregulation of phosphorylated Akt Enhancement of autophagy [103]
Cyclooxygenase 2	Poxviruses	Impaired anti-viral antibody production leading to increased viral replication [104]

## Abbreviations

Head and Neck Squamous Cells Carcinoma (HNSCC); Oncolytic viruses (OVs); Squamous cell carcinoma (SCC); Herpes simplex virus type 1 (HSV-1); Coxsackievirus and adenovirus receptor (CAR), Intercellular Adhesion Molecule 1 (ICAM-1)); granulocyte-macrophage colony-stimulating factor (GM-CSF); cytokine release syndrome (CRS); Tumor Necrosis Factor (TNF); pathogen-associated molecular patterns (PAMPs); Interferon (IFN); retinoic acid-inducible gene 1 (RIG-1), Janus kinase(JAK)/ Signal Transducer and Activator of Transcription(STAT); protein kinase R (PKR); Talimogene Laherparepvec (T-VEC), Food and Drug Administration (FDA); immune checkpoint inhibitors (ICIs), cutaneous squamous cell carcinoma (CSCC); 5-fluorouracil (5-FU); non-small cell lung cancer (NSCLC). Histone deacetylase inhibitors (HDIs), valproic acid (VPA); hydroxamic acid (SAHA); trichostatin A (TSA); Immune checkpoint blockade (ICB); tumor microenvironment (TME); oncolytic vaccinia virus (VACV).
